# Video-assisted mediastinoscopic transhiatal esophagectomy combined with laparoscopy for esophageal cancer

**DOI:** 10.1186/1749-8090-5-132

**Published:** 2010-12-31

**Authors:** Bin Wu, Lei Xue, Ming Qiu, Xiangmin Zheng, Lei Zhong, Xiong Qin, Zhifei Xu

**Affiliations:** 1 Department of Cardio-Thoracic surgery, Changzheng Hospital, Second Military Medical University, 415 Fengyang Road, Shanghai, 200003, PR China; 2 Department of Minimally Invasive Surgery, Changzheng Hospital, Second Military Medical University, 415 Fengyang Road, Shanghai, 200003, PR China

## Abstract

**Background:**

Minimally invasive transhiatal esophagectomy for esophageal cancer includes mediastinoscopic and laparoscopic transhiatal esophagectomy. It is inadequate in both two techniques. It is impossible to dissect the lower esophagus with single mediastinoscopy or the upper and middle esophagus with single laparoscopy. We use mediastinoscopy combined with laparoscopy to dissect the whole esophagus and stomach including lymph node dissection. In addition, laparoscopic gastric mobilization leads to less trauma than an open gastroplasty.

**Methods:**

40 cases of video-assisted mediastinoscopic transhiatal esophagectomy were performed and divided into two groups.32 patients were received surgical therapy of single mediastinoscopic esophagectomy with open gastroplasty in group A, while 8 patients were received surgical therapy of mediastinoscopic esophagectomy combined with laparoscopic lower esophageal and gastric dissection in group B. The perioperative complications were recorded.

**Results:**

Video-assisted mediastinoscopic transhiatal esophagectomy was performed successfully both in group A and B. It suggested that mediastinoscopy combined with laparoscopy be better than single mediastinoscopy because of less blood loss, less pain, shorter ICU stay and complete lower mediastinal lymph nodes resection.

**Conclusions:**

Video-assisted mediastinoscopic transhiatal esophagectomy combined with laparoscopy is a safe and minimally invasive technique with whole esophagus and mediastinal lymph node dissection in the clear visualization of the mediastinum, reducing the abdominal trauma.

## Background

Since the late 1980 s, minimally invasive surgical technique has been widely used in diagnosis and treatment of chest disease. The overall advantages of minimally invasive surgery are to complete the same operation through small incision avoiding the trauma of open operation. Traditional operation for esophageal carcionma requires thoracotomy and laparotomy, which is one of the most complex operations in gastrointestinal surgery. The trauma is large and the morbidity of surgical complications is high. So the surgeons are searching for a minimal invasive operative method instead of traditional esophagectomy.

The basic uses of mediastinoscopy include mediastinal mass biopsy, lymph node biopsy for the diagnosis. With the development of endoscopic technology, the applicative area of mediastinoscopy expanded. By now video-assisted mediastinoscopy can be used for the separation of esophageal tumor. Esophagectomy via mediastinoscopy was firstly reported by Buess[1] in 1990. The advantage of video-assisted mediastinoscopic transhiatal esophagectomy is not only to avoid thoracotomy and reduce bleeding compared with traditional transhiatal esophagectomy, but also to resect mediastinal lymph node thoroughly. Also, laparoscopic techniques have developed rapaidly in recent years, which can be used to mobilize the stomach and to mobilize the lower esophagus via hiatus. A combination of mediastinoscopy and laparoscopy could be used for complete esophagectomy and reconstruction of digestive tract, which can replace the single mediastinoscopic esophagectomy to reduce trauma and postoperative complications[2].

## Methods

### General data

From March 2004 to November 2008, 40 cases of video-assisted mediastinoscopic esophagectomy were performed in our Department. All the surgical treatments were completed by the same group of surgeons. All the patients had been diagnosed and staged preoperatively by endoscopy with biopsy, X-ray of the digestive tract with barium swallow, CT scan of the chest and abdomen, and ultrasound of the neck. In addition, all patients completed respiratory function tests and two-dimensional cardiac ultrasound examination to determine the surgical risk. The 40 paitients were divided into two groups(Table 1). 32 patients were received surgical therapy of video-assisted mediastinoscopic esophagectomy with open gastroplasty(Group A).8 patients were received surgical therapy of video-assisted mediastinoscopic esophagectomy with laparoscopic lower esophageal and gastric dissection (Group B). Ethical approval was given by the medical ethics committee of Changzheng Hospital. All patients signed informed consent before treatment.

**Table 1:** Patient characteristics and grade of esophageal carcinoma in two groups

	Group A	Group B	Total
Gender			
Male	20	8	28
Female	12	0	12
Age			
Mean ± SD (yrs)	59.3 ± 10.1	67.0 ± 7.1	65.5 ± 9.4
Range (yrs)	42-78	55-73	42-78
Carcinoma location			
Upper	16	2	18
Midddle	14	4	18
Lower	2	2	4
p- stage			
T			
T is	0	1	1
T 1a	8	2	10
T 1b	10	3	13
T 2	8	1	9
T 3	6	1	7
T 4a and T 4b	0	0	0
N			
N0	22	7	29
N1a	8	1	9
N1b	2	0	2
N2 and N3	0	0	0
M			
M0	32	8	40
M1	0	0	0
H			
H1	32	8	40
H2	0	0	0
G			
Gx	5	0	5
G1	9	3	12
G2	16	4	20
G3	2	1	3
G4	0	0	0
TNM-stage			
0	0	1	1
Ia	7	3	10
Ib	14	1	15
II	10	3	13
IIIa	1	0	1
IIIb and IV	0	0	0

### Operation method

#### video-assisted mediastinoscopy with open gastroplasty (Group A)

Two surgeons(cervical team) performed upper and middle esophageal mobilization with the video-assisted mediastinoscope via a left cervical approach while other two surgeons (abdominal team)prepared the lower esophageal and gastric dissection via the traditional transabdominal approach. The mediastinoscope was inserted carefully from anterior diastema of the vertebral column and pushed gently into the mediastinum. The pultaceous connective tissue close to the posterior side of esophageal was dissected bluntly using a special aspirater with electric coagulation. The main gross lymphatic and anathreptic blood vessels were safely exposed and coagulated with 5-mm Laparoscopic Curved Shears (LCS, Ethicon Endosurgery, LLC). Other small vessels were coagulated with the special coagulator that suctioned simultaneously. The mediastinoscopy was gradually moved forward, the farthest to 15 cm. A piece of gauze was filled in to oppress the operating field for hemostasis before drawing out the mediastinoscopy. Then the mediastinoscope was inserted into the tracheoesophageal diastema to separate the anterior side of esophagus by the same way. The mediastinoscope was turned right and left to dissect both sides of esophagus gently with the LCS. The upper and middle esophagus was completely dissected when meeting the gauze. The upper and middle thoracic paraesophageal lymph nodes were exposed and dissected. During this procedure, the abdominal team prepared the gastric mobilization. After conventional gastroplasty, the diaphragmatic hiatus was enlarged. The operator from abdominal team inserted his left index finger to separate the lower esophagus blindly to meet the mediastinoscope inserted from cervical team. Then the dissection of the whole esophagus was completed. The esophagus was cut in the abdomen. A 20-cm long bandage was binded with a 30-cm long traction suture line which was sutured to the stump of the esophagus. The esophagus was then pulled through from the mediastinum to the neck. The bandage was pulled into the mediastinum for oppression in 5 minutes and then pulled through from the neck. The mediastinoscope was inserted again to confirm hemostasis and to dissect the remnant lymph nodes. An end-to-end cervical esophago-gastric anastomosis was then completed.

#### video-assisted mediastinoscopy combined with laparoscopy (Group B)

Mediastinoscopy in patients with postural and operation techniques was described in the preceding paragraph. The abdominal team prepared video-assisted laparoscopic lower esophageal dissection and gastric mobilization. The limbs of the diaphragmatic crura and two vagus nerves around the lower esophagus were incised by LCS. Aftrer enlarging the diaphragmatic hiatus, the laparoscope was then inserted and pushed gently into the lower mediastinum. The laparoscope was gradually moved forward to meet the mediastinoscope directly. During the mediastinal dissection, the lymph nodes and soft tissue were dissected. Gastric tubulization was completed along the greater curvature, using a 45 mm EndoGIA(ETS 45, Ethicon Endosurgery, LLC) and the esophagogastric junction was then dissected. One end of a 30-cm long suture line was sutured to the fundus of stomach, then the other end was sutured to the stump of the esophagus. The esophagus was then pulled through from the mediastinum to the left cervical part. The mediastinoscope was inserted to dissect the remnant lymph nodes. An end-to-end cervical esophago-gastric anastomosis was then completed.

## Results and discussion

Video-assisted mediastinoscopic transhiatal esophagectomy was performed successfully both in group A and B. There was no hospital death in both two groups. The results were listed in Table 2 and suggested that mediastinoscopic transhiatal esophagectomy combined with laparoscope be better than single mediastinoscopic transhiatal esophagectomy because of less blood loss, less pain, shorter ICU stay and complete lower mediastinal lymph nodes resection.

**Table 2:** Perioperative clinical data in two groups

	Group A	Group B
Conversion to open surgery	0	0
Average operative time(min)	180	220
Average mediastinoscopic time	108	100
Average abdominal time	80	120
Average total blood loss(ml)	218	100
Average number of lymph node dissection	12	15
Intraoperative splenic rupture*	1	0
Pulmonary infection	1	0
Mediastinal chyle leakage^▵^	1	0
Recurrent laryngeal nerve injury	2	0
Anastomotic leakage	3	1
Pain(visual analog score)	8	4
Mean ICU stay(day)	2.2	1.2
Mean postoperative hospital stay(day)	11.6	10.6

*Splenectomy was performed in one patient because of intraoperative splenic rupture with 600 ml blood loss. ^▵^Mediastinal chyle leakage was recorded in one patient. The amount of mediastinal chyle was totle 900-2000 ml/d. At last a lower ligation for thoracic duct was performed through right thoracotomy after inefficacious conservative treatment of one week.

Esophageal cancer surgery is complex. The morbidity of postoperative complications is high[3]. There are two main operative approaches in traditional open esophagectomy. One is transthoracic esophagectomy(TTE), another is transhiatal esophagectomy(THE). At the early 90 s esophagectomy has been developed on the basis of the concept of minimally invasive surgery. Several laparoscopic approaches for the esophageal cancer have been proposed including video-assisted thoracoscopic surgery(VATS)[4], laparoscopic transhiatal esophagectomy[5], Mediastinoscope-assisted transhiatal esophagectomy (MATHE)[1] and Video-assisted Ivor-Lewis esophagectomy[6]. In recent years thoracoscopy associate with laparoscopy or mediastinoscopy associate with laparoscopy as the surgical approaches have been reported[7].

THE is advantageous because it avoids one-lung ventilation(OLV) and does not need change the body position. The risks and limitations of THE are bleeding, tracheal injury and recurrent laryngeal nerve injury due to blind manipulation of the esophagus and the inability to perform lymph node dissection. So THE is only for T1 cancer[8]. Bumm[9] reported the technique of MATHE and concluded that mediastinoscopy through left cervical approach was very helpful for dissection of the upper esophagus and trachea. But It was impossible to dissect the lower esophagus. It also allowed biopsy of several mediastinal lymph nodes, with the advantage of protecting the recurrent laryngeal nerve because the mediastinal structures can be visualized directly. Bumm[10] compared 47 patients who underwent mediastinoscopic esophagectomy with 61 patients who underwent esophageal pull-off approach during the same period. The rates of pneumonia, hypopnoea, cardiac complications and recurrent laryngeal nerve injury were lower in the mediastinoscopy group. In our study, two cases of recurrent laryngeal nerve injury occurred. The two cases were both upper thoracic esophageal cancer with T3 period of the tumor stage. When dissecting the tumor we found the ambient connective tissue adhered to the tumor closely. So we also removed adhesive connective tissue surrounding the tumor including the recurrent laryngeal nerve, resulting in postoperative hoarseness. Postoperative mediastinal chyle leakage was recorded in one patient. We neglected to check the thoracic duct during the surgery. The amount of mediastinal chyle was totle 900-2000 ml/d. At last a lower ligation for thoracic duct was performed through right thoracotomy. In another patient the thoracic duct injury was found during the surgery and the distal thoracic duct was then clipped by titanium clamp. We believe that the thoracic duct would not be damaged if the loose tissue around the esophagus could be bluntly dissected.

De Paula[11] and Swanstrom[5] reported laparoscopic whole esophagectomy including laparoscopic gastric dissection and transhiatal esophageal dissection. But the laparoscopic approach are inadequate for the upper third of the esophageal dissection because of the upper mediastinal structures and the length of laparoscope. The upper lymph node metastases are also cannot be reached.

Mediastinoscopy combined with laparoscopic surgery had been reported by Bonavina[2] in 2004. This procedure avoided the disadvantage of single mediastinoscopic or laparoscopic esophagectomy. Video-assisted mediastinoscopy dissected the middle and upper thoracic esophagus under direct vision while laparoscopy dissected the lower esophagus. The whole esophagus can be dissected without dead ends. All lymph nodes of esophageal bed were visible and could be resected synchronously. We performed video-assisted mediastinoscopic transhiatal esophagectomy with laparoscopic lower esophageal dissection and gastric mobilization compared with the single mediastinoscopic esophagectomy. The mediastinal structures like trachea and totle mediastinal lymph nodes could be visualized directly under the endoscopic images. It was possible to dissect lymph nodes completely using mediastinoscope and laparoscope. The laparoscopic gastric dissection was also safer and more accurate than open gastroplasty, reducing the morbidity of intraoperative splenic rupture. We also found that level of pain after laparotomy was higher than that after laparoscopy. The patients after laparotomy were afraid of cough and expectoration, thereby increasing the incidence of pulmonary complications. However, non-randomized controlled study of this research can not draw meaningful conclusions.

It is difficult for both mediastinoscopy and laparoscopy to resect the eminence lymph nodes completely. So the preoperative CT scan is important to exclude from the patients with fusion of eminence lymph nodes. Besides, whether lymph node dissection should be required is still in dispute. Some scholars[12] believe that esophageal cancer with lymph node metastasis is a holistic system disease. Lymph node dissection can not improve the survival rate of esophageal cancer. Lymph node dissection should be palliative by sampling and pathologic examination. However, most authors[13] believe that esophageal resection with simultaneous lymph node dissection of esophageal bed is conducive to long-term survival. We agree with the latter.

## Conclusions

Video-assisted mediastinoscopic transhiatal esophagectomy without lung collapse is more suitable for the patients with poor lung function. But single mediastinoscopic esophagectomy is disadvantageous because it is difficult to resect the whole esophagus and mediastinal lymph nodes. Mediastinoscopy combined with laparoscopy is a safe and effective minimally invasive technique to solve the problem. In addition, the number of cases is not enough for statistical significance. Our work is still in progress.

## Competing interests

The authors declare that they have no competing interests.

## Authors' contributions

BW and LX helped with design of the study, data interpretation and co-wrote the manuscript. MQ and XZ helped with surgical techniques, collection of data and data analysis. LZ and XQ participated in study design, gathering patient information and performed the tables. ZX carried out study design, coordination and made main correction of the manuscript according to the reviewers' suggestions. All authors read and approved the final manuscript.
